# Instance-based generalization for human judgments about uncertainty

**DOI:** 10.1371/journal.pcbi.1006205

**Published:** 2018-06-04

**Authors:** Philipp Schustek, Rubén Moreno-Bote

**Affiliations:** Center for Brain and Cognition and Department of Information and Communications Technologies, Pompeu Fabra University, Barcelona, Spain; Harvard University, UNITED STATES

## Abstract

While previous studies have shown that human behavior adjusts in response to uncertainty, it is still not well understood how uncertainty is estimated and represented. As probability distributions are high dimensional objects, only constrained families of distributions with a low number of parameters can be specified from finite data. However, it is unknown what the structural assumptions are that the brain uses to estimate them. We introduce a novel paradigm that requires human participants of either sex to explicitly estimate the dispersion of a distribution over future observations. Judgments are based on a very small sample from a centered, normally distributed random variable that was suggested by the framing of the task. This probability density estimation task could optimally be solved by inferring the dispersion parameter of a normal distribution. We find that although behavior closely tracks uncertainty on a trial-by-trial basis and resists an explanation with simple heuristics, it is hardly consistent with parametric inference of a normal distribution. Despite the transparency of the simple generating process, participants estimate a distribution biased towards the observed instances while still strongly generalizing beyond the sample. The inferred internal distributions can be well approximated by a nonparametric mixture of spatially extended basis distributions. Thus, our results suggest that fluctuations have an excessive effect on human uncertainty judgments because of representations that can adapt overly flexibly to the sample. This might be of greater utility in more general conditions in structurally uncertain environments.

## Author summary

Are three heavy tropical storms this year compelling evidence for climate change? A suspicious clustering of events may reflect a real change of the environment or might be due to random fluctuations because our world is uncertain. To generalize well, we should build a probability distribution over our observations defined in terms of latent causes. If data is scarce we are forced to make strong assumptions about the shape of the distribution ideally incorporating our prior knowledge. In our task, human behavior is consistent with probabilistic inference but reveals a tendency to generalize based on observed instances enhancing the effect of random patterns on behavioral judgments. The decreased reliance on available constraints through prior knowledge corresponds to a dominance of bottom-up sensory information. Maintaining a balance with expectation-driven top-down information is crucial for proper generalization. Our work provides evidence for the necessity to include graded instance-based generalization into the mathematical formulation of cognitive models. The investigation of the determinants and neural substrates of this inferential bias is expected to give insights into the richness but also fallibility of human inferences.

## Introduction

Determining from limited data when observations reflect a consistently appearing pattern or when they are merely the result of randomness is important to faithfully represent the environment (e.g. [1]). Suppose you want to assess the skill of a dart player in throwing darts at the bullseye (center) of the board. For a single bad throw, it is hard to discern whether it was due to bad luck or to the general inability of the player. For several throws, however, the dispersion of the darts around the center should more closely reflect the skill of the player.

To represent uncertainty of our knowledge in this and more general situations, normative considerations suggest that an agent should explicitly represent knowledge as probability distributions instead of point estimates [2,3]. Several studies have shown that under certain conditions humans behave as if the uncertainty about a task-relevant variable was available to them as a distribution over its possible values [4,5].

For instance, judging the skill of the dart player corresponds to estimating the spread of the distribution around the observed values. This requires constraining structural assumptions about the ‘shape’ of the underlying probability distribution (e.g. a parameterized function such as a Laplacian or Gaussian). However, it is generally unknown what assumptions are used by humans when dealing with uncertainty. Ideally, previous knowledge about the data generation process, such as an expectation for the darts to cluster around the center corresponding to the goal in the example, is incorporated. As opposed to visuo-motor uncertainty [6], there is little evidence for the shape of inferred trial-by-trial perceptual representations in the small sample limit. In several previous studies such as cue combination [7], distributional estimates are taken to be normally distributed. While this may be justifiable under certain conditions [8], we challenge the general validity of this assumption.

To generalize from sparse data, one inevitably must make assumptions about the distribution. In other words, we have to choose a suitable model for probabilistic inference. In the most elementary case, probability density must be assigned to the vicinity of an observed point in some internal psychological space defining a metric of similarity between possible occurrences [9].

In doing so, weak assumptions give more freedom to the observed instances of the data to determine the inferred distribution. The resulting generalizations are similarity-based and have been used to explain certain characteristics of how humans learn continuous functions [10,11]. Similarly, such instance-based or exemplar methods were suggested to describe the representations that underlie human categorizations [12–14]. If such inferences are formulated in probabilistic terms, this is commonly implemented by nonparametric methods, such as kernel density estimation [15].

Stronger assumptions, on the other hand, may allow for more powerful generalizations [16,17] if they are based on appropriate prior knowledge about the task structure [18]. Correspondingly, a more restricted class of parametric probability distributions is used. The function learning literature refers to the more constrained case as rule-based [19] because humans appear to learn explicit functions of some family, such as polynomials [20,21]. Similarly, strong assumptions can be incorporated into models of categorization by positing a prototype for each category [22]. Critically, we emphasize that inferential methods are not limited to the extremes of strong and weak assumptions but may exist as combinations along a continuum [22,10,23].

Here we asked what kind of internal structural assumptions humans employ to generalize from sparse observations. Human participants are asked to quantify uncertainty about future events by estimating the dispersion of a normally distributed random variable. Although the instructions and the framing of the task suggested a simple, centered, unimodal, bell-shaped distribution, human behavior was not consistent with structural assumptions based on a close to normal probability distribution. Instead, human behavior was better explained by instance-based generalization whereby observed samples were used to build an internal representation of the underlying probability distribution, not necessarily unimodal or symmetric. The resulting internal representation is a mixture of several components and hence less sparse than necessary. Our participants demonstrated faithful trial-by-trial estimates of uncertainty which are suggested to originate from internal uncertainty representations, as the opportunity to learn suitable stimulus-response associations from feedback was avoided in our task design [3]. All alternative heuristic explanations proved insufficient to explain the complex and consistently accurate estimates. Hence, our results support the notion that approximate probabilistic processing underlies behavior.

## Results

**Fig 1:**
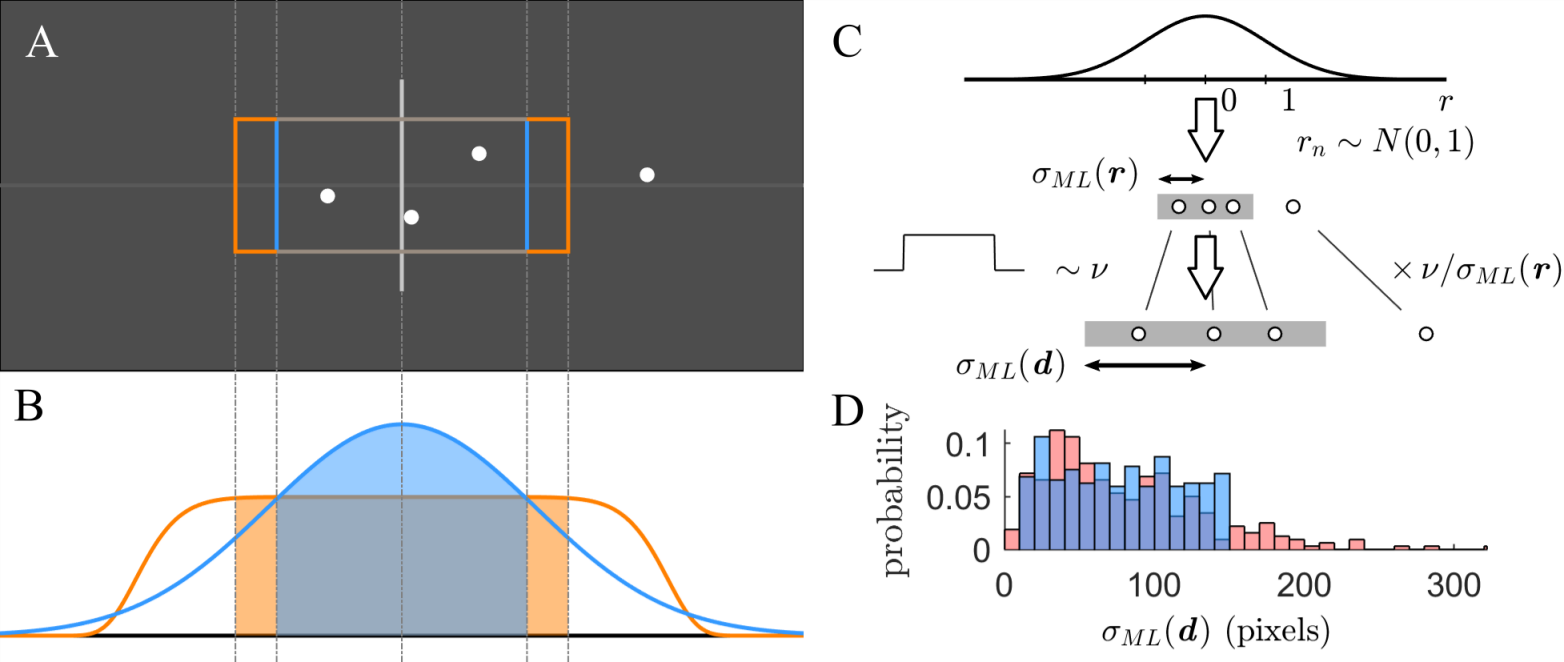
Human participants perform a task consisting in estimating the dispersion of future events based on a few observations. **(A)** Schematic of one trial of the task. Participants were asked to judge the unknown accuracy of a “dart player” to hit the center of the board (gray rectangle). Based on the four observed “darts” (white dots), participants must predict where future darts might strike the board. Specifically, participants were asked to capture 65% of all future imaginary darts by adjusting the width of the rectangular frame (colored frames, see below). Only the horizontal dispersion of the dots is relevant to estimate the accuracy of the dart player, while vertical displacements are added just to improve visibility of the samples. ***(B)*** Based on the observed samples, the participant might infer a predictive probability distribution over the position of the next sample. Two hypothetical predictive distributions are shown, representing different structural assumptions about how the samples might have been generated, corresponding to maximum likelihood estimation based on a Gaussian distribution (blue) or a generalized normal distribution with shape parameter *p* = 10 (orange) (see Methods). Based on the predictive probability distribution, the participant can set the frame’s width so that it matches the target percentage of 65% (colored frames in panel A). Note that for the assumption of a generalized normal distribution, the posterior is more sensitive to data points far from the center and hence a larger frame is chosen. **(C)** The horizontal positions of the points with respect to the center were generated as follows. First, all samples ***r*** *= (r*_1_,…,*r*_4_) were generated independently from a standard normal distribution. Second, the samples were scaled by the factor *ν*/*σ*_*ML*_(***r***), where $\sigma_{ML}(r)=\sqrt{1/N\sum r_{n}^{2}}$ is the maximum likelihood estimator (MLE) for a normal distribution centered at zero and *ν* is drawn from a uniform probability distribution over the range of [10,140] pixels. The scaled samples ***d*** = *ν*/*σ*_*ML*_(***r***) ⋅ ***r*** feature a MLE given by $\sigma_{ML}(d)=\sqrt{1/N\sum d_{n}^{2}}=\nu$. This method allows choosing any desired distribution of *σ*_*ML*_(***d***) by setting *ν* correspondingly. **(D)** Histogram of *σ*_*ML*_(***d***) across 320 trials (blue). For comparison, the red histogram indicates the results for a sample scaling ***d*** = *ν* ⋅ ***r*** without normalizing by *σ*_*ML*_(***r***). Both samples have a comparable mean, but the red distribution features few but extremely outlying values, which are avoided by our scaling method.

We asked human participants to estimate the dispersion of future events from a small sample by indicating a range in which they predicted 65% of all future events to fall. The task instructions alluded to judging the ability of a dart player to hit the target based only on the outcome of previous attempts (Fig 1). More specifically, participants were asked to judge the unknown accuracy of a “dart player” to hit the center of the board (Fig 1A). On a given trial, of a total of 320 trials, the participants are shown four points representing the “darts” thrown by one unobserved player of unknown accuracy to hit the center of the board. Based on the four observed “darts”, participants must predict where future darts might strike the board. Specifically, participants were asked to capture 65% of all future imaginary darts from the same unobserved player by adjusting the width of the rectangular frame of size 2*y* symmetrically about the center (*y* is the horizontal, one-sided distance of the lateral borders of the rectangle to the center). Only the horizontal dispersion of the dots is relevant to estimate the accuracy of the dart player, while vertical displacements are added just to improve visibility of the samples. The choice of 65% is convenient as it does not depend on an accurate estimate of the distribution’s tail and conveniently allows to examine a limiting case of instanced-based generalization. Participants were informed that they would see a new player of unknown and fixed accuracy to hit the center in every trial, that there would be just as many amateur as expert level players and that the order of appearance is unpredictable.

Ideally, this task could be accomplished by inferring the dispersion of the generative distribution which in accordance to the task and its instructions was chosen to be Gaussian. Based on the observed samples, a probabilistic agent would infer a predictive probability distribution over the position of the next sample to accurately estimate the size of the frame that would capture 65% of the imaginary darts thrown by the very same dart player with the same abilities. Inference requires the specification of a generative model of the observed data. However, the actual generative model in the environment, controlled by the experimenter, and the model that the agent uses for inference are generally different. Nevertheless, in order that inference is optimal for this task, the agent’s probabilistic model needs to match the generative process. Exploiting knowledge that a normal distribution *d*_*n*_ ~ *N*(*μ* = 0,*σ*) centered at zero is responsible for the *N* = 4 observations ***d*** = (*d*_1_,…,*d*_*N*_), estimation of the predictive density *p*(*x*|***d***) over an unseen event *x* amounts to inference of the only unknown quantity, the standard deviation *σ*, parameterizing the zero mean Gaussian. Maximizing the likelihood function *p*(***d***|*σ*) with respect to *σ* yields $\sigma_{ML}={\left(1/N\sum_{n=1}^{N}d_{n}^{2}\right)}^{1/2}$ which corresponds to the expression for the standard deviation with a known mean of zero. The predictive distribution may be directly based on the specific value determined by maximum likelihood estimation (MLE) *p*(*x*|*σ* = *σ*_*ML*_(***d***)), which is illustrated in Fig 1B. However, given the observations it is not possible to determine *σ* with certainty. The maximum likelihood estimator *σ*_*ML*_ and the number of observations *N* can only be regarded as sufficient statistics for *σ*.

The Bayesian treatment explicitly acknowledges this uncertainty by computing the posterior distribution *p*(*σ*|***d***) over possible values of *σ*.

$$p(\sigma |d)\propto \prod_{n=1}^{N}N(d_{n}|0,\sigma )\cdot p(\sigma )$$ (1)

Additionally, this requires the specification of the prior distribution *p*(*σ*) which is part of the agent’s subjective knowledge. However, to be task-optimal, it must equal the actual distribution over *σ* in the environment, i.e. the base rate at which the hidden variable *σ* occurs. For this task, it should be a uniform distribution over the range of [0,140] pixels. To then predict the probability of the next event at position *x* given ***d***, *σ* has to be marginalized out. The predictive distribution results from the probabilistic model *N*(*x*|0, *σ*) weighted by the posterior over *σ*.

$$p(x|d)=\int_{0}^{\infty }N(x|0,\sigma )\cdot p(\sigma |d)d\sigma$$ (2)

More generally, the predictive distribution *p*(*x*|***d***) corresponds to the belief about future events after observing data ***d***.

Now, we turn to the problem of how the agent might set the frame in a principled way based on the estimated predictive probability distribution. For a given setting of the rectangular frame *z*, one can determine the fraction of future events within that interval, the capture probability *c*, by calculating the integral

$$c(z)=\int_{-z}^{z}p(x|d)\;dx$$ (3)

More generally, the inferred distribution in (3) provides an objective to determine the response *y* (half-frame size) on a trial-by-trial basis. To match the target probability of 65%, the frame size *z* should be optimized such that the capture probability matches the target probability (Fig 1B). In other words, the response *y* is the optimized frame size that matches

c(y)=0.65 (4)

This inference procedure of a normal distribution whose width is assumed to vary parametrically across trials is devised as a reference model (benchmark) for comparison with behavior. It follows the inference procedure of Eqs (1 and 2) and assumes a uniform prior over the range of [0,140] pixels corresponding to the task instructions. The Bayesian benchmark model was chosen as reference for motivational feedback and bonus payments to incentivize engagement in the task (see Methods).

However, to generate the data ***d*** that was presented to our participants, we used a slightly modified sampling scheme which reduces response noise and keeps outlying conditions to a minimum translating into improved discriminatory power for model comparison (see Methods). This was achieved by renormalization of the raw samples ***r*** (Fig 1C). Therefore, a draw *ν* from the uniform distribution over the desired range of dispersions directly determines the sufficient statistic *σ*_*ML*_. Omitting sample renormalization instead corresponds to sampling from a Gaussian whose width parameter *σ* is drawn from a uniform distribution. This would have led to a long-tailed *σ*_*ML*_(***d***) distribution with undesirable properties (s. Fig 1D) which is avoided by our approach.

**Fig 2:**
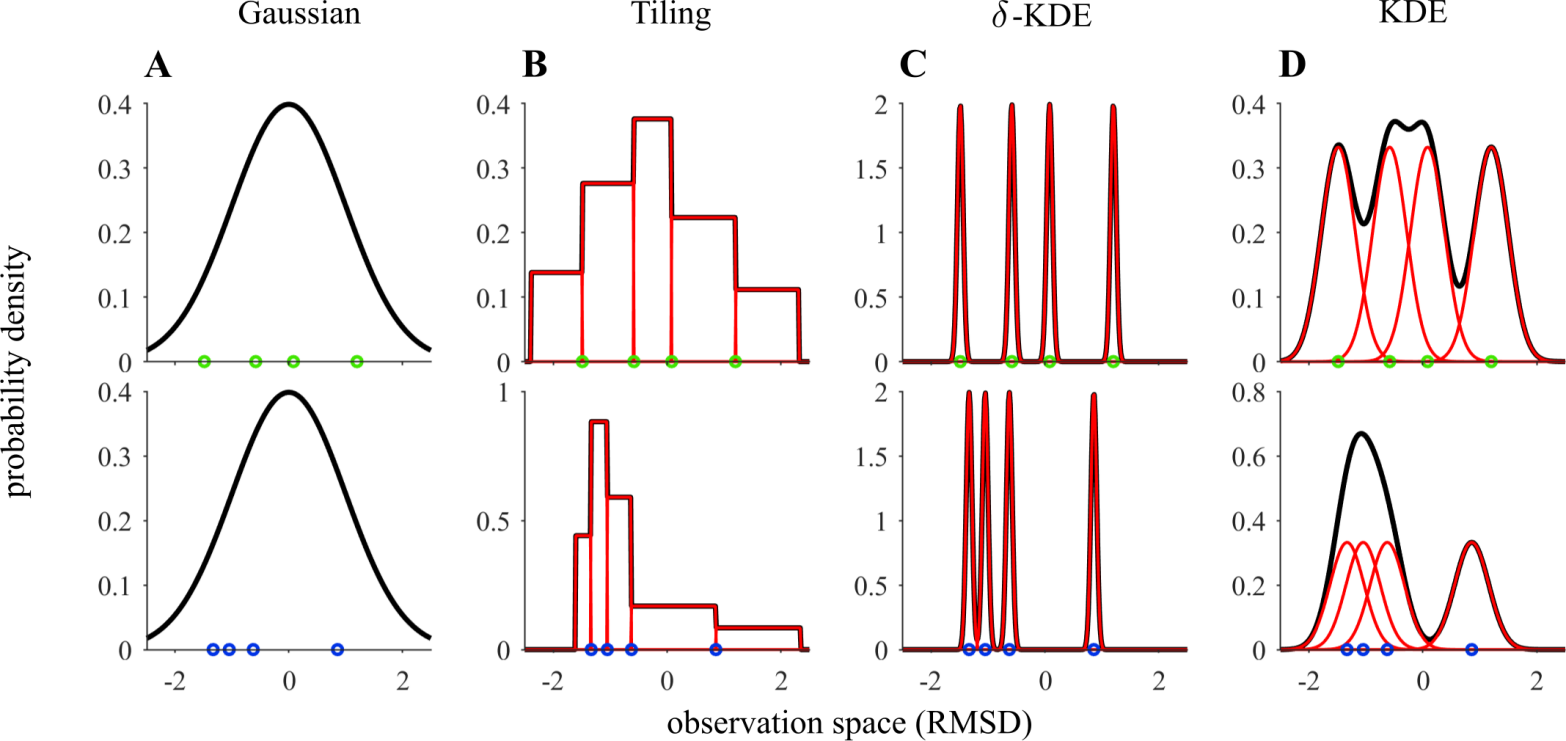
Generalization beyond the observed sample is governed by the parametric assumptions of the distribution. Each row shows examples of probability densities (black lines) for a different sample (green and blue dots, four observations) in units of its root mean squared deviation (RMSD). **(A)** A zero-centered unimodal Gaussian distribution is used to account for the whole sample. All point positions ***d*** = (*d*_1_,…,*d*_4_) enter via the estimated standard deviation parameter, *σ*_*ML*_(***d***) (RMSD), determined by probabilistic inference. Whereas for instanced-based generalization the sample points effectively enter as parameters themselves. **(B-D)** Different additive basis distributions (red) can be used to cover the observation space. The tiling model covers the space with adjacent non-overlapping uniform basis distributions resulting in a compressed distribution around spatially proximal points **(B)**. Additionally, models can be constructed from simpler components by centering a Gaussian kernel on each observation (see Methods). In the limit of vanishing kernel widths **(C)** there is no generalization beyond the sample while for larger widths **(D)** a smoothed density over the whole domain is obtain due to overlapping basis distributions.

The goal of the study is to determine which inductive biases participants employ for generalization and whether that conforms to the structural assumptions suggested by the framing of the task. More specifically, we attempted to distinguish between inference of a centered, unimodal, bell-shaped distribution, such as a Gaussian (Fig 2A), and variants of instance-based generalization (Fig 2B–2D) which make only very few assumptions about the distribution to be inferred.

We will test several models of the latter class to obtain more information about the specific characteristics of the internal representations. The tiling model constructs a normalized histogram under the constraint that an observed point only exhibits a local effect on the constructed density by tiling the domain into non-overlapping basis distributions (Fig 2B). Similar representations were, for instance, suggested to underlie the representation of visuo-motor errors [24]. The degree of generalization critically depends on how far away from a sample’s position the inferred density is affected [25]. Therefore, we use a kernel density estimation method [22] with Gaussian, and hence spatially extended, basis distributions. The width of these basis distributions critically governs the locality of the influence of the sample on the internal representation and will be inferred from behavior. For very narrow Gaussians (*δ*-KDE, Fig 2C) generalization is weak whereas large and overlapping kernels indicate stronger generalizations beyond the sample (Fig 2D). We furthermore investigated whether participants might derive their behavior from an internal representation of a probability distribution. Alternatively, any measure that correlates with the dispersion to be estimated might serve to inform behavior. These heuristics are primarily chosen to facilitate processing and not to achieve a more accurate representation of the environment. Our task allows explicit testing of some heuristic short-cuts to the task.

### Faithful tracking of trial-by-trial uncertainty

**Fig 3:**
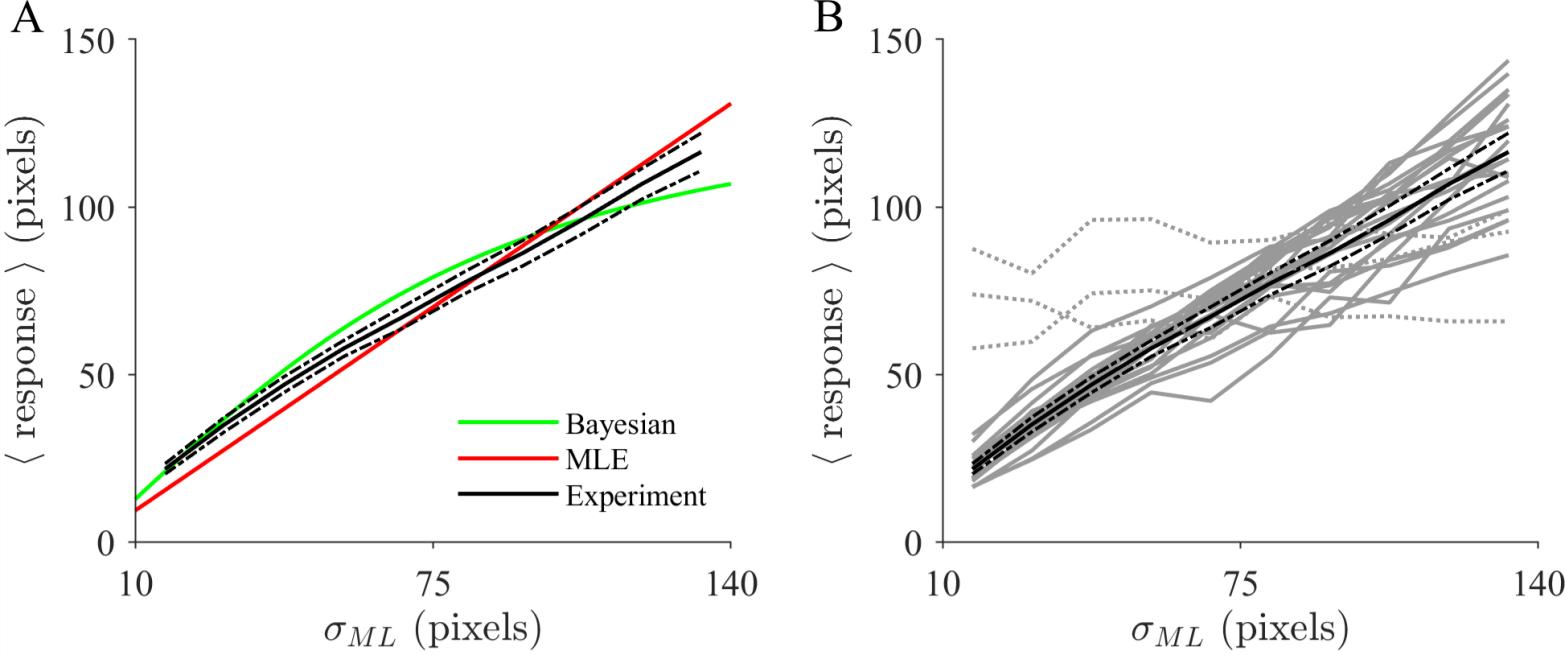
Human behavior closely tracks trial-by-trial uncertainty of future events. **(A)** Mean responses across participants plotted as a function of the MLE of the sample, *σ_ML_(**d**)*, in ten equally spaced bins (black; error bars, 95% CI). Basing behavior on a Gaussian estimated by ML (red, *N(x|0,σ_ML_(**d**))*) results in responses proportional to the estimate. The prior distribution that is assumed by the devised Bayesian benchmark model (green) biases responses towards intermediate values (see Methods). **(B)** Individual response curves of all 23 participants tested (gray lines). Three participants displaying poor compliance with the instructed task (dotted) were excluded from further analysis. The average across the remaining participants is superimposed (black).

First, we tested whether participants demonstrate the ability to faithfully estimate the dispersion of the centered normal distribution assumed to be responsible for the observations. The MLE of the Gaussian, *σ*_*ML*_ (Fig 3A, red), is the sufficient statistic to inform the optimal response (green).

The averaged mean response across participants (black) is related to the ML approach in a approximately linear relationship (Methods). Assuming that participants use the Gaussian distribution for inference (Methods, normal model) yields good predictive performance and accounts for a substantial amount of the variance (regression, cross-validated median *R*^2^ = 0.80, 95%-CI (0.73,0.82), across participants). Hence their judgments correlated tightly with the uncertainty about the abilities of the supposed dart players. Such uncertainty tracking is also apparent on an individual participant level (Fig 3B) (cross-validated median *R*^2^ ranging from 0.47 to 0.93). On average, the responses appear to be systematically biased toward intermediate values with respect to the ML approach (Fig 3A, red) resembling the effect of a prior distribution (green) incorporating knowledge about the range of dispersions across trials. To quantify this effect, we used two variants of a model (Methods, Weighting model) that effectively determines whether judgments may still be predicted well if they are assumed to be proportional to the estimated dispersion (Eq 7). However, this was found to be strongly inferior to a linear relationship (Methods, Eq 5), even on an individual level (cross validation log likelihood (CVLL) difference Δ ≥ 20 for 12 participants, Δ ≥ 10 for 17 participants).

### Evidence for an internal trial-by-trial objective

Next, behavior is examined with respect to the objective participants were instructed to obey. Namely, if their estimates are quantitatively accurate and correspond to the 65% target percentage. For independent trials, participants must infer the dispersion anew on each trial. Inferring a probability distribution over future events allows behavior to be derived from a principled trial-by-trial objective regarding the target percentage (see Fig 1B and Methods, Eqs 3 and 4). By construction, our task objective demands a quantification of the relative frequency of all future events and was intended to require participants to approximate distributional estimates.

**Fig 4:**
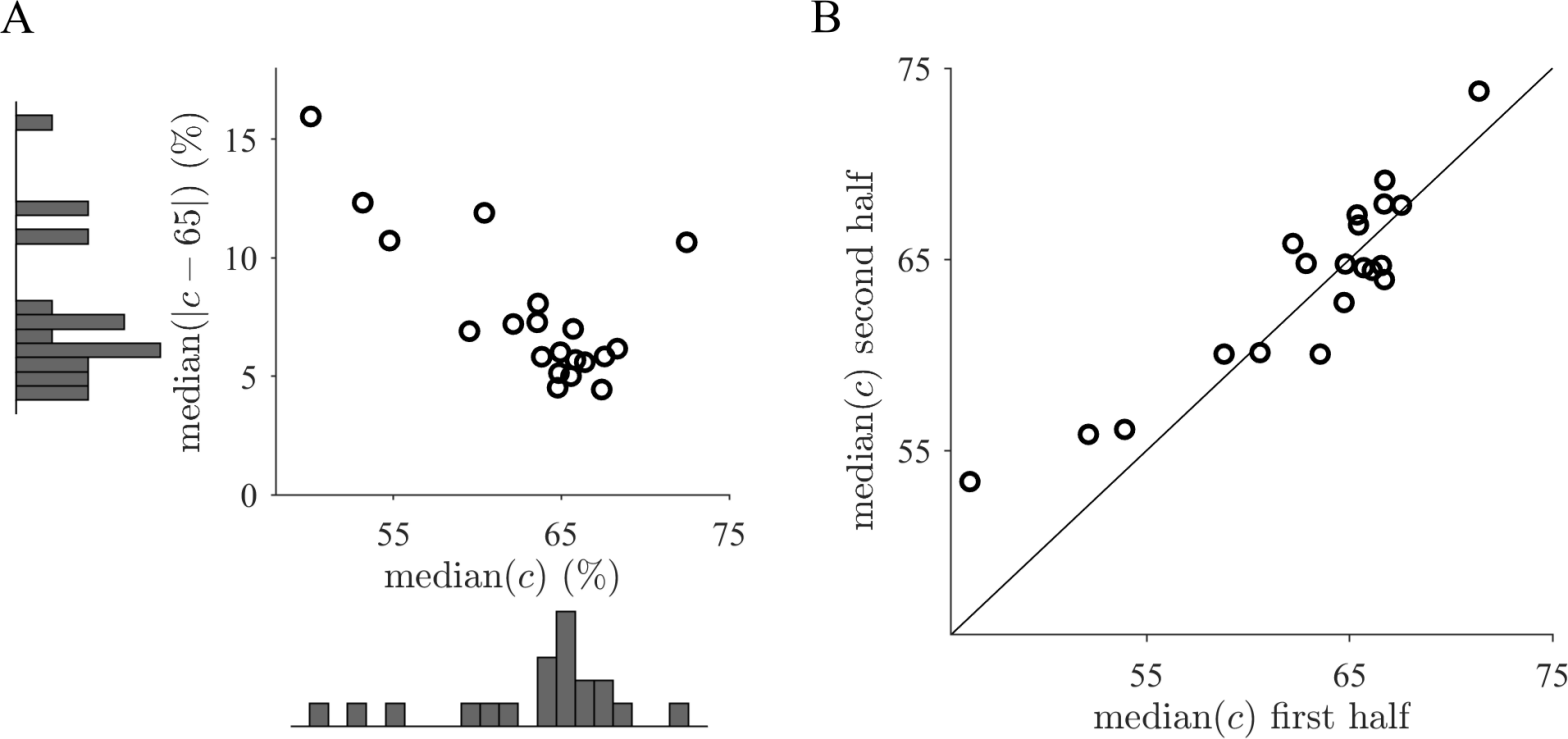
Behavior is consistent with participants possessing a subjective but well calibrated trial-by-trial internal objective that remains stable over the experiment. (**A**) Across trials participants tend to comply well to the objective despite per trial deviations due to systematic biases and response noise, as the capture percentage *c* is typically around the target value 65% (vertical axis) and the median deviance is relatively small (horizontal axis). Histograms correspond to marginal distributions. (**B**) Participants display stable behavior throughout the experiment, as they do not appear to adjust their responses closer to the task objective over time. Median capture percentages *c* are calculated separately for the first and second halves of the experimental session.

To examine how well participants performed with respect to the devised optimal inference strategy, we calculated the capture percentage by evaluating (Eq 3) with respect to the optimally inferred probability distribution (Eqs 1 and 2). The distribution of the per participant median capture percentage across all trials is clustered close to the target of 65% (Fig 4A). In this measure opposing deviations cancel, so that it evidences an overall compliance to the target percentage across all trials. The median across participants is close to the target percentage, which indicates that participants quantify uncertainty in a quantitatively similar manner as the probabilistic benchmark model. The median of the absolute deviation per response is 6.54% (95% CI, (5.83,7.28) %) with respect to the external objective of the task. However, it is possible that behavior has been produced from an internal objective (see Eq 4) in which the percentage is matched much more closely to 65%. There are at least two contributions that inflate the deviation from the external measure (Fig 4A). First, there is intrinsic response noise which would even occur for fixed stimuli on the screen, e.g. through motor-related variability. Second, there are deviations due to mismatched inference with respect to our benchmark model [26]. The latter are deterministic and the result of e.g. different prior knowledge from the one assumed by our benchmark model. Altogether, the median absolute deviation (Fig 4A) is a conservative upper bound estimate for an internal trial-by-trial objective of the capture percentage such that the quantitative match with the target percentage can be considered high.

If participants did not possess an internal trial-by-trial objective, they could instead associate stimuli with suitable responses by a learning a behavioral function. Next, we tested whether behavior is consistent with this alternative approach, which should result in across-trial and feedback dependencies. Even though barely informative, the feedback may have been used to adjust behavior. Remarkably, however, the median capture percentage appears not to adjust closer to the target percentage as indicated by similar values calculated separately for the first and second half of the experimental session for each participant (Fig 4B). The absolute difference of the median capture deviation is small and not significantly different from zero (right-tailed Wilcoxon signed rank test, *p* = 0.48) despite the fact that the trial-averaged feedback about the capture percentage in the experimental session may have allowed to derive some global adjustments. Accordingly, too high a capture percentage on average should subsequently lead to the choice of smaller response frames. Hence, a decrease of the feedback error would be expected over time. The results, on the other hand, suggest that participants did not even use feedback to calibrate their probability estimates. We also confirmed that the previously presented feedback about the capture percentage did not influence behavior (regression, exceedance probability 2.04 ⋅ 10^−4^ compared to baseline model, see Methods). Similarly, no considerable dependencies across trials were found (Methods). Consequently, it appears unlikely that the feedback scheme had an important influence on behavior.

Overall, participants typically predict the dispersion of future darts in a quantitatively accurate manner. They appear to have relied on an internal trial-by-trial objective regarding the target percentage as they largely conform to trial independence, feature stable processing across time and virtually ignore feedback. This is consistent with internal probabilistic processing.

### Systematic deviations from inference of a Gaussian

Thus far, behavior appears to be close to the optimal inference strategy defined by the benchmark model, but we have also observed deviations (Figs 3 and 4A). If behavior follows from inference of a normal distribution, it can only depend on the sample via the sufficient statistic, $\sigma_{ML}(d)=\sqrt{1/N\sum_{n}d_{n}^{2}}$. This means that the squared position of each point should contribute equally to the final estimate. We tested this with a weighting model that generalizes *σ*_*ML*_ by assigning a tunable weight *ω*_*n*_ to each input depending on its excentricity, $\sqrt{1/N\sum_{n}\omega_{n}d_{n}^{2}}$. Excentricity refers to the distance from the center irrespective of the side where the sample occurs.

**Fig 5:**
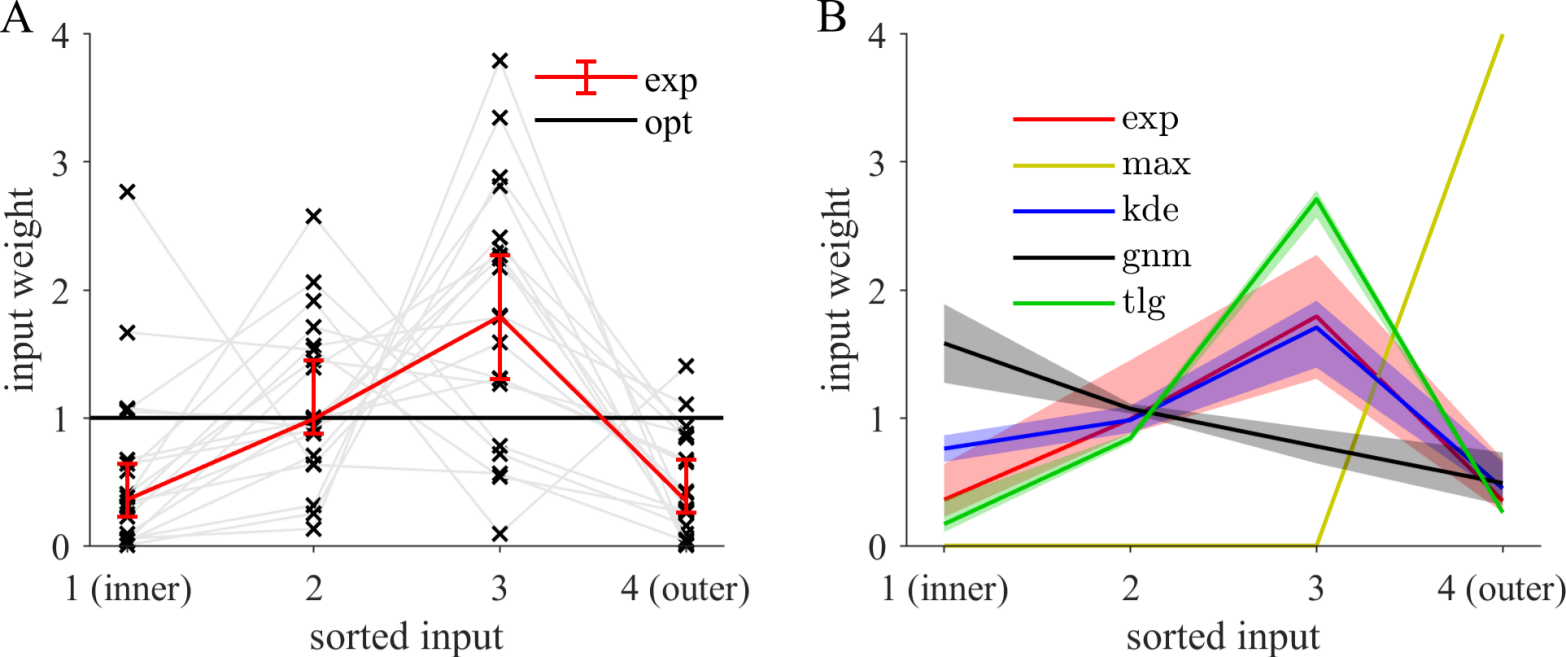
The weighting pattern of the observed samples deviates from inference of a close-to-normal distribution and matches kernel density estimation (KDE). Evaluation of the normalized weights *ω_n_* of the weighting-model $\hat{S}(d)=\sqrt{1/N\sum_{n}\omega_{n}d_{n}^{2}}$ as a generalization of the MLE of a zero centered Gaussian. The points are indexed according to their distance from the center. **(A)** Input weight that each participant (gray lines) assigns as a function of the weight index. If participants followed optimal MLE based on a Gaussian centered at zero, all input weights should be equal (black line). Fitting of the weighting model (see Methods) shows a systematic deviation of the across participant median (red, error bars, 95% CI). Participants tend to overweigh the third most extreme value compared to the others. **(B)** Among all models tested, only KDE (blue) qualitatively matches the characteristics of the experimental weighting pattern (red, same as panel A). The other models fail to capture the behavioral weighting pattern (fits of the weighting model to the other indicated models’ output). Model abbreviations: kde—kernel density estimation, tlg—tiling, gnm—generalized normal, max–maximum.

**Fig 6:**
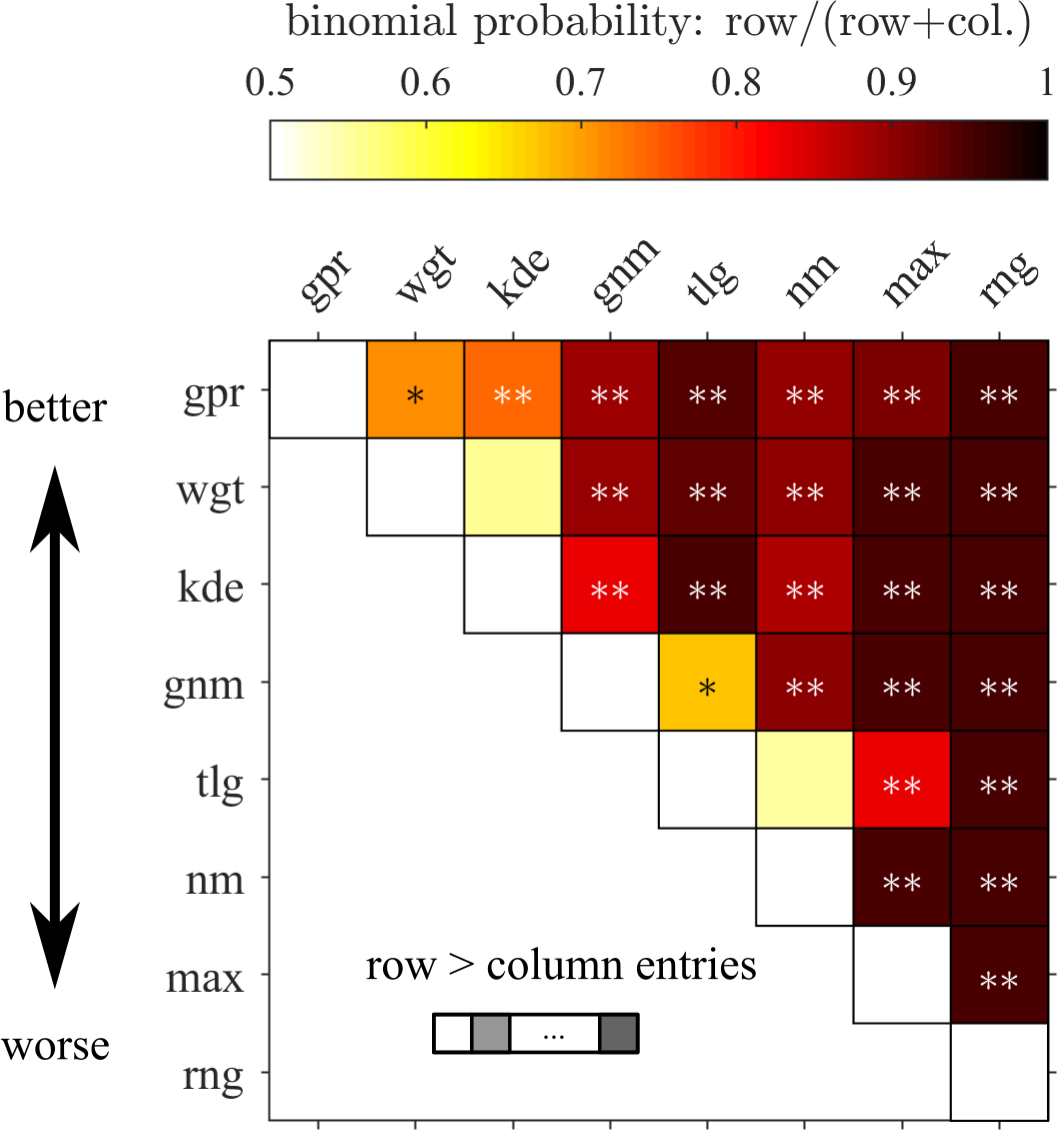
Pairwise model comparison evidences an inclination to resort to instance-based generalization, indicating that fluctuations have a profound effect on the inferred representations. Summarized results of a hierarchical Bayesian model comparison procedure that estimates probability distributions over models. Pairwise comparisons (each square) are performed to evidence relative differences in prediction for models with different features. The color code over each square shows estimates of the parameter of the binomial distribution governing the probability by which the model indexed by the row is more likely than the one indexed by the column. This corresponds to the expectation value that a given model is considered responsible for generating the data of a randomly chosen participant. Superimposed are large differences of the exceedance probability ($*\hat{=}(0.99>p_{exc}\geq 0.95)$; $**\hat{=}p_{exc}\geq 0.99$) which quantifies the belief that the row model is more likely to have generated the data of a randomly chosen participant compared to the column model. Model abbreviations: gpr—Gaussian process regression, wgt—weighting, kde—kernel density estimation, gnm—generalized normal, tlg—tiling, nm—normal, max—maximum, rng–range.

Experimentally, the weights of the individual points tend to take unequal values when we index them with respect to their distance from the center (Fig 5A). Participants put more emphasis on the third most excentric point and down-weigh the first and the fourth point. We also tested whether other models of behavior, such as the KDE model, are able to reproduce this pattern (Fig 5B). For that purpose, we used those fitted models to generate surrogate responses for every actual experimental response. Subsequently, for the comparison, the weighting model was fitted to the surrogate responses. In the following, models will be compared by both the (i) weighting pattern (Fig 5B) as well as their (ii) overall ability to predict behavior (Fig 6). Consistent with the weighting pattern observed in our data, the normal model (nm) is far from providing the best predictions of behavior. This can be seen from the pairwise model comparison matrix (Fig 6). There the binomial probability that the model indexing the row (vs. the model indexing the column) is more likely to account for the data of a randomly chosen participant is depicted as color code. Additionally, entries with high exceedance probabilities are considered significant (Methods) and marked with asterisks. For instance, the comparison between the weighting model in row (wgt) to the normal model in column (nm) shows that the latter is clearly rejected (*p*_*exc*_ > 0.999). Beyond the group level, the normal model can be decisively ruled out individually for many participants despite the fact that generally different participants are best described by different models.

We tested whether generalizations of the Gaussian can account for the systematic deviations that were observed before. The generalized normal model (gnm) allows for more freedom in the representation of the inferred density through a shape parameter governing its kurtosis (see Fig 1B) by generalizing the square in the exponential function to other powers than two leading to an unequal weighting pattern of the samples (Fig 5B). This model predicts significantly better than the Normal-model (Fig 6, *p*_*exc*_ > 0.999) by making use of the additional shape parameter to represent heavier tailed distributions (quartiles across participants *Q* = (0.79,1.24,1.68)). Heavier tailed distributions discount outlying and enhance the influence of inlying points on judgments (Fig 5B, black line). The experimental pattern (red) is not matched well suggesting that it does not reflect how participants behave. In addition, the weighting model still outperforms the generalized normal model (Fig 6).

### Simple heuristics are poor predictors

We determined above that responses are on average relatively close to optimal but that the finer-grained behavioral patterns are inconsistent with inference of a Gaussian. That raises the question whether simpler, heuristic strategies that unequally weigh sample information might offer a better account of behavior.

We first tested the established heuristic models that use perceptually simple statistics and only a subset of the available information. The maximum model (max) only depends on the most excentric point which leads to a weighting pattern (Fig 5B, yellow) which is highly inconsistent with the experimental one (red). The participants’ weighting is more balanced and typically features weights smaller than four (normalization to number of sample points). The range model (rng) is based on the sample's range and predicts worse than the maximum model (Fig 6). On the group level, both are clearly refuted by all other models.

Another heuristic strategy is attending to just one point when sorting them according to their excentricity. In particular, the third most excentric point is important as it closely corresponds to the target percentage of 65% on the sample and is the response in the limiting case of pure instance-based generalization (see *δ*-KDE model, Methods). Participants typically take all point positions into account. The four unnormalized weights (*w*_1_,…,*w*_4_) are significantly different from zero for the respective number of (14, 20, 20, 19) of all 20 participants (Weighting model, 10000-fold permutation test, *p*-value threshold 0.05). Furthermore, for each individual at most one weight is non-significant showing that it is not an effect of grouping. Consistent with integration of the whole sample, the maximum of the normalized weights is considerably lower than four (Fig 5A).

Altogether, this is evidence that among all participants few exploit heuristics. The clear majority however resorted to some more sophisticated weighting inconsistent with the simple heuristics tested.

### Behavior relies on instance-based generalization

So far, participants appear to violate the assumptions of a close to Gaussian distribution centered at zero that was suggested by the task instructions and the dart metaphor. Alternatively, the probability distribution to be inferred may be directly constructed from the observed instances by imposing only minimal structural constraints on the data. That corresponds to the assumption that the sample is representative of the unknown population to be estimated.

Our tiling model (tlg) implements such an approach with spatially confined basis distributions. It places a uniform distribution in between observations and hence the resulting density is increased around clusters and reduced elsewhere (Methods). It adapts to the fluctuations which are present in the sample. Consequently, the target capture percentage of 65% is by construction very close to the third most excentric point. As a result, this model emphasizes the third most excentric point (Fig 5B, green) and thus captures an important characteristic of behavior (red).

The kernel density estimation (KDE) model uses Gaussian basis functions to implement instance-based generalization. It centers a Gaussian distribution on each data point and thus assigns density to its vicinity depending on the standard deviation parameter. The experimental weighting pattern (black) is closely captured by KDE (Fig 5B, blue). It is very successful at predicting behavior and superior to both the normal and the generalized normal model considered before (Fig 6). The small and insignificant difference of the model probability (Fig 6, wgt vs. kde) indicates that KDE predicts on a similar level as the weighting model even though the latter has more adaptable parameters and thus may be considered more flexible. The weighting model does not explicitly construct a probability density but can be viewed as a functional approximation that can capture similar dependencies of behavior on the sample.

In summary, participants do not sufficiently exploit the structural constraints suggested by the task but instead give more freedom to the specific instances of the observations to determine their responses. The tendency to assume that even small samples are representative of the population could be well captured by nonparametric kernel density estimation.

### Inferred representations feature overlapping and redundant kernels

Probability distributions over perceptual variables should be embedded in the context of more general knowledge of the task’s context. From a causal inference perspective, they should be attributed to the causal variables already known to exist. Treating all observations as if they originate from their own cause, i.e. as new causal variables, makes purely nonparametric methods seem of limited applicability in wider contexts. In this sense, KDE itself may be considered a heuristic approach as it largely ignores prior (structural) knowledge. Examining the inferred representations, we argue here that there is reason to believe that behavior is not purely nonparametric but can rather be conceived of as an instance-based modulation, or bias, to causal inference.

**Fig 7:**
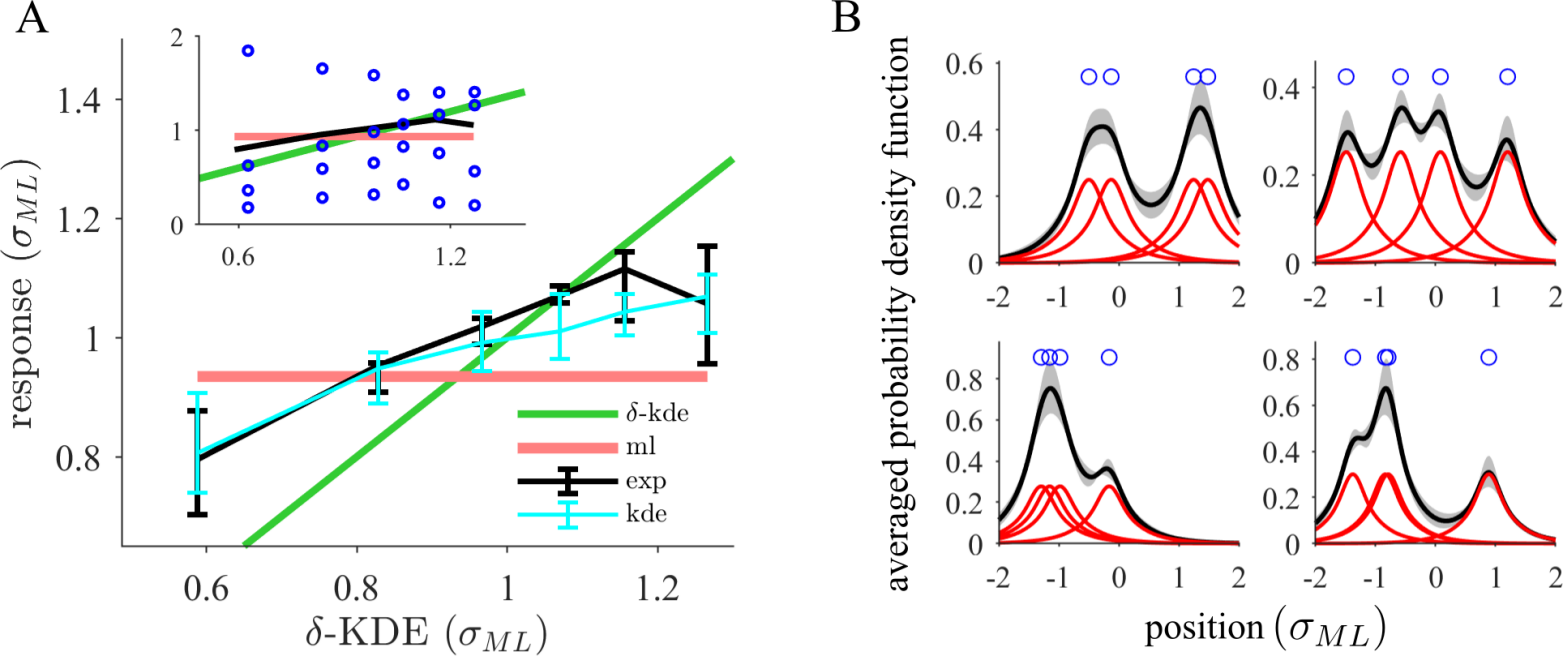
Strong generalization is consistent with the possibility of integrating prior knowledge about the task structure. **(A)** Responses (black) show higher consistency with inference of a single Gaussian than with approaches generalizing only weakly beyond the sample such as *δ*-KDE (limit of vanishing kernel widths; third most excentric sample point). The plot shows aggregated (median across participants, 95% CI) bin medians of the responses (normalized by *σ*_*ML*_) and the fitted KDE model (cyan) as a function of the *δ*-KDE output (approximately equally filled bins). By construction, inference of a Gaussian results in a horizontal line (red) while *δ*-KDE (green) yields a linear function of slope one. The experimental curves are less steep indicating a rather moderate instance-based modulation compared to a Gaussian model. The inset is a zoomed-out version additionally showing the relationship of the responses to the distribution of sample points (median of absolute value within each bin). **(B)** The KDE model infers internal distributions that are smoothed and spatially extended around the sample points. The mean probability density function across participants (black, 95% CI) is shown for four different samples (blue circles). The inferred density is smooth featuring fewer modes than the number of basis distributions (red curves). This is a consequence of the large fitted Gaussian kernel widths which lead to substantial overlap of the basis distributions.

If we infer very narrow kernel functions for our participants that indicates that there is very little generalization from the sample. For close to orthogonal kernel functions with virtually no overlap (e.g. delta-distributions) the output reduces to a mere counting of observations. First, we tested how strong this instance-based bias is on the level of raw responses by comparing them to the predictions of *δ*-KDE (Fig 7A). Both axes are normalized to the MLE, *σ*_*ML*_, of the sample (i.e. the draws from the standard normal distribution, see Methods). All responses are plotted as a function of the *δ*-KDE output. Thus, by construction, predictions of *δ*-KDE (green) itself follow the unity line while predictions of inference using a Gaussian likelihood function follow a constant line of slope zero (red). Values of the optimal benchmark model would fluctuate because of varying prior beliefs that average to a constant independent of the sample given the MLE. The slope of a linear function fitted to the experimental responses is far from one as expected from *δ*-KDE (Fig 7A, regression, median slope across participants 0.27, 95%-CI (0.24,0.38)). As opposed to the *δ*-KDE model, the KDE model (cyan) can predict the behavioral pattern (black) well because its kernel width parameter takes large values (Fig 7B, red) (median across participants 0.40, 95%-CI (0.35,0.59), in units of *σ*_*ML*_). Participants capture a varying number of points with the response frame (Fig 7A, inset) which is only possible if the constructed density is a non-local function of the specific sample configuration on the screen. This slope pattern is not entirely inconsistent with inference of a Gaussian likelihood function as responses actually vary around its value as a function of the sample configuration. On the contrary, the normal model reaches high predictive performance in absolute values as shown before. However, additional to the responses derived from Gaussian inference, there are subtle instance-based variations which can be captured by the KDE model. At the level of the responses, behavior may be understood as inference of a normal distribution that is modulated by KDE.

Interestingly, KDE predicts behavior significantly better than the tiling model (Fig 6). The main difference is that the tiling model relies on spatially confined basis functions while Gaussian kernels are spatially extended. The weighting pattern shows that the tiling model (Fig 5B, green) overweighs the third most excentric point even more than behavior (red). The tiling model too closely resembles the purely instance-based approach of *δ*-KDE while behavior is not so strongly influenced by the third most excentric point. Of all models tested KDE (blue) best captures the weighting pattern (Fig 5B) because the large kernel width exhibits a non-local effect so that the positions of all points influence judgments leading to a more balanced pattern.

A large kernel width makes spatially extended basis distributions overlap (Fig 7B, red). Accordingly, we typically find fewer than four modes in the inferred densities of the participants (median of the per participant mean across trials 2.0375,95% *CI* = (1.50,2.27)). Thus, increasing the kernel width may be understood as a reduction of the effective number of components in the mixture distribution as measured by the number of modes (Pearson correlation coefficient, *ρ* = −0.95, *p* = 6.01 ⋅ 10^−11^). Our data requires the KDE model to perform close to a regime where it must approximate inference of some smooth distribution which is closer to unimodal. Despite being the best approximation explored, it is nevertheless possible that the inference method used by our participants is structurally more constrained than KDE and uses some prior knowledge of the task structure.

From a representational point of view, the large overlap of the basis distributions (Fig 7B, red) is a rather redundant and thus inefficient way of representing the whole distribution. For a large degree of overlap, several kernel functions could be well represented by a single kernel function whose free parameters are tuned to accommodate all their contributions. Bayesian nonparametric mixture models [27] can effectively reduce the number of redundant mixture components and minimize shared responsibility to account for the data points. The number of components can adapt to the position and number of data points in the sample. It gives less freedom to the data than KDE but implements soft and gradual constraints towards sparsity. A preference for sparser or denser representations can be specified by a prior. Likewise, prior knowledge such as a zero-centered population may be included in this way. We suggest this as a connection to theoretical principles.

We found that participants show different preferences for instance-based generalization. The average number of modes of the inferred densities according to the KDE-model almost covers the full range of possible values (minimum 1.01, median 2.04, maximum 3.63, across participants). Even with wide kernels, KDE is limited in its ability to represent unimodal near-Gaussian distributions. Correspondingly, the difference in predictive performance (CVLL) between the KDE and the normal model is larger for smaller kernel widths (linear correlation coefficient, *ρ* = −0.54,*p* = 0.0075). Consistent with previous results, the slope in Fig 7A decreases with the kernel width (Pearson correlation coefficient, *ρ* = −0.66, *p* = 7.30 ⋅ 10^−4^). The determinants of the participants' preferences are unclear from this experiment. We remark however, that participants who infer more redundant densities tend to respond faster (Spearman correlation coefficient, *ρ* = 0.35,*p* = 0.064) although the result does not reach significance.

In summary, using KDE we found very wide overlapping kernels leading to densities which could be more sparsely represented. This hints at a more sophisticated inference approach than pure instance-based generalization. It may be considered a modulation of causal inference by a kernel-based approach. We suggest a connection to Bayesian nonparametric methods in statistics that allow to incorporate prior knowledge and sparsity constraints.

### Explanation close to ceiling level

There are many possible ways in which this task might be approached by our participants. Thus, we attempt to estimate an upper bound of the predictable structure in the data regardless of how the task was solved by the participant. Gaussian process regression (GPR) is used to find a low-bias functional approximation between input ***d*** and behavior *y*. Hence, if a model reaches similar predictive levels, this is indication that it captures the most relevant computational operations. GPR is indeed found to be the best model (Fig 6) on the group level. However, the differences to the KDE-model are not disconcertingly large (median CVLL difference across participants, 13.3 dHart, 95%-CI, (−3.1, 23.5) dHart). Overall, KDE can predict on a comparable level as GPR. This is remarkable as for interpretable models, all factors need to be specified explicitly. For instance, even motor related variations with ***d*** would have to be incorporated. Moreover, as probability densities are high dimensional and subjective, the achieved match is not trivial.

## Discussion

This study attempted to elucidate how sensory representations of uncertainty are constructed from sparse data. We have described a new experimental task that allows us to measure quantitative judgments of uncertainty in response to a noisy stimulus with high precision. We find that (1) participants give faithful judgments about uncertainty on a trial-by-trial basis which are irreducible to simple heuristics. (2) Their behavior is not in agreement with the structural assumptions of a Gaussian suggested by the framing of the task. Instead, according to their behavior, participants are biased to judge the sample as representative of the population and that random fluctuations in the sample will reproduce in the long run. A connection to Bayesian nonparametric models is suggested to model this inclination towards instance-based generalization. (3) Furthermore, behavior is consistent with the idea that participants internally represent the variable of interest probabilistically as a normalized distribution over its possible values.

The idea that perception constitutes some form of (probabilistic) inference process was suggested long ago [28]. It has a particular appeal for deriving subjective estimates of uncertainty as it emerges naturally from the knowledge representation itself, i.e. from the posterior distribution, without requiring a meta-representation [29–31].

Experimentally, one must elicit the read-out of a suitable summary statistic of the sensory representation. In previous work, participants are typically asked to report their confidence that the latent variable to be inferred lies beyond some fixed decision boundary [32]. Instead, we allowed participants to freely estimate the dispersion of the inferred density. There is virtually no demand on working memory and participants do not need to resort to language to perform the task. Both aspects are believed to be critical for promoting rational behavior [33]. In addition to being intuitive, this task requires an ability to deal with uncertainty to construct an internal objective on a trial-by-trial level regarding the target percentage.

Critically, this task was designed to minimize sensory and motor noise to obtain a sensitive probe of behavioral variations of dispersion estimates. As opposed to prior work, e.g. using the random dot motion stimulus [34,35], here mainly the task-relevant stimulus dimensions drive behavior. This study more specifically investigates the process of density estimation that is embedded in other (hierarchical) tasks. Previously, several studies tested how multiple inferred sensory representations are combined. The reliability based weighting of conflicting cues from different modalities suggests that distributional estimates are provided by each modality [7]. Another study also supplied evidence by means of a dot cloud [4] but assumed that participants know that the observations are normally distributed when making inference. Many previous studies made the strong assumption that participants know the generative process of the task. Very often it is chosen to be a normal distribution [34,36]. It may be a reasonably good proxy to model cognitive processes for simple, nonlinear and low-dimensional stimulus tasks with abundant evidence. However, we challenge the adequacy for inference in complex environments or sparse observations. These assumptions evade the deeper question of choosing a suitable model that the agent faces. In hierarchical models and depending on context, the upper levels provide constraints as to what the important causal factors are. We framed the task by alluding to a commonly known random process of throwing darts conforming to prior structural assumptions of a centered, unimodal and bell-shaped distribution that is close to Gaussian.

Nevertheless, we find that most participants fall short of these assumptions but rather give systematically biased estimates. Because of the low number of samples, our task allows testing what inductive biases [37] participants exhibit. They appear to give more freedom to the model’s structure to adapt to the sample. Thus, their judgments seem to assume that fluctuations in the sample are representative of the population [38]. However, we found evidence that their inferences are somewhat more constrained than purely instance-based estimates leading to potentially sparser representations. We propose to view this in the framework of Bayesian nonparametric mixture models [27,39] which may infer the appropriate complexity for each sample based on a prior expressing a preference for the sparsity of the final estimate (the number of components). In this context, the bias towards instanced-based generalization can be considered a prior that favors more complex solutions. This is reminiscent of findings in the literature where human abilities to learn functions are described by a hybrid of instance-based, nonparametric and rule-based, parametric approaches [10]. We believe that these ideas merit further exploration and extension to more complex causal structures and tests with different sample sizes. For a simple, monocausal generative model, as in our case, we would expect that the number of components of the internal representations becomes sparser as more data is provided, because there are no more features to be captured. Furthermore, it is intriguing to ask if a similar probabilistic inference perspective may be helpful to explain the decreased reliance on outlying evidence in a decision task between two stimulus categories [40]. This was originally attributed to robust estimation which may be seen as inference of a mixture distribution in which additional components are used to explain observations that are far too outlying to be considered part of the main process. Correspondingly, their contribution to an estimate of the main process would be reduced.

We can only speculate about the reasons behind this inductive bias. First, it might be due to considering the cost of computing [41] in an attempt to simplify judgments. However, we found a tendency towards more complex representations whereas sparser representations are typically believed to be more economical. For example, decomposing high-dimensional objects such as continuous probability density functions of human visuo-motor errors into simple non-overlapping (uniform) basis distributions was suggested to be a solution to complexity by obtaining a sparser representation [6]. Instead, we speculate that the bias towards instanced-based generalization might be related to structural uncertainty about the causes of their observations. Structural uncertainty has been shown to lead to model-free learning [42]. Similarly, a sensitivity to small alterations in the task setting has been found to affect optimality of behavior [43]. Furthermore, we might be equipped with a more fundamental bias to perceive causes behind patterns even for little evidence [44].

By construction, our task objective only applies to a normalized distribution over future outcomes regardless of its functional shape. Various studies have claimed that internal processing is probabilistic or at least demonstrated a “lower bound for the sophistication of confidence evaluation” [45]. Typical approaches derive an optimal solution to the task and show that behavior is reasonably close to it. However, strong claims require preconditions [46] such as testing alternative models [47] for non-trivial optimal processing. We do not claim optimal processing but emphasize systematic deviations that nevertheless might originate from internal probabilistic computations. Often as in our case, a clearly suboptimal strategy yields near-optimal results.

In fact, instead of a trial-by-trial objective for the target percentage derived from a density estimate, a learnt stimulus-responses mapping might be used instead. Our task design minimized the possibility to optimize a reward measure through trial-and-error over trials by omitting informative feedback. Consequently, the chances of acquiring a stimulus-response mapping are minimized. Furthermore, simple heuristic approximations [48] to behavior have been ruled out explicitly. Additionally, we found that the implementation of instance-based generalization by KDE is within reasonable bounds of an estimate of the predictable structure in behavior [46] suggesting that we have captured the important computations.

Ultimately, the degree to which claims to probabilistic processing seem substantiated depends on the propensity to belief that the task could alternatively be solved by a well-tuned mapping or heuristic estimator acquired prior to the experiment. This task is rather artificial, and humans are seldom prompted to state or give error intervals in terms of percentages. Accordingly, the situations to learn from are sparse. Uncertainty about (latent) variables is rarely made explicit, especially in numerical terms, but rather implicitly used by the agent to integrate and update beliefs. Generally, there is little information about the frequency with which events happen in our world across instances of the same situation. Even though learning calibrated mappings from specific situations is in principle possible, it is highly uneconomical and thus regarded unlikely. Likewise, it seems unrealistic that evolutionary training across generations has provided us with well-tuned heuristics for specific situations such as this task. After all, we deem it more plausible to assume that most participants estimated some (approximate) probabilistic distribution to derive their judgments.

In conclusion, our results suggest that human judgments about uncertainty are guided by an internal probabilistic objective. However, there is a tendency to identify fluctuations in the sample as representative for judgments about the population. This may be captured by a representation endowed with a preference to adapt overly flexibly to the observed instances.

## Materials and methods

### Ethics statement

Comité Ético de Investigación Clínica, Parc de Salut MAR, Barcelona Spain, 2013/5464/I, titulado “Del laboratorio a la calle: El impacto de la integración multisensorial en la vida cotidiana”. Written informed consent was obtained from all participants.

### Sampling scheme to generate observations

On each of the 320 trials, the horizontal positions of the points with respect to the center were generated as follows (Fig 1C). First, always *N* = 4 sample values ***r*** = (*r*_1_,…,*r*_4_) are independently drawn from a standard normal distribution *r*_*n*_ ~ *N*(0,1). Second, the samples were scaled by the factor *ν*/*σ*_*ML*_(***r***), where $\sigma_{ML}(r)=\sqrt{1/N\sum r_{n}^{2}}$ is the maximum likelihood estimator (MLE) for a normal distribution centered at zero of the samples ***r*** and *ν* is drawn from a uniform probability distribution over the range of [10,140] pixels. The scaled sample ***d*** = *ν*/*σ*_*ML*_(***r***) ⋅ ***r*** always has a MLE given by $\sigma_{ML}(d)=\sqrt{1/N\sum d_{n}^{2}}=\nu$. This method allows choosing any desired value of *σ*_*ML*_(***d***) by setting *ν* correspondingly. Setting *σ*_*ML*_(***d***) directly, which is the main determinant for inference, has the advantage that observations ***d*** and the MLE *σ*_*ML*_(***d***) take less extreme values which translates into increased numerical stability for model comparison. Defining an explicit latent *σ*-variable over a finite range instead would have led to a long-tailed *σ*_*ML*_(***d***) distribution with undesirable properties (s. Fig 1D). The ability to tell apart models with similar predictions is enhanced if response noise and outlying conditions are kept at a minimum.

However, because of this way of generating the dots, the optimal inference model with respect to the actual generative model in the environment is not readily defined. Nevertheless, participants do not know these alterations to how the dots were generated. The best they can do is to follow the instructions and their prior knowledge suggested by the dart metaphor to explain the data. We do not define the optimal model with respect to the generative model in the environment. Instead, we define it as an optimal inference strategy based on a normal distribution whose width varies parametrically across trials. It follows the inference strategy of Eqs (1 and 2) and assumes a uniform prior over the range of [0,140] pixels. As this prior arguably matches the task instructions it was chosen as a basis for our Bayesian benchmark model and the feedback in the experiment.

### Participants and experimental procedure

In total 23 participants (15 female, 8 male) were recruited mainly among students from the Pompeu Fabra University in Barcelona. We accepted all healthy adults with normal or corrected to normal vision. We obtained written confirmation of informed consent to the conditions and the payment modalities of the task. The training and the experimental session were carried out on a single appointment that nominally lasted 75 min. First, participants read detailed written instructions of the task. In a brief training session, they were given 40 trials to familiarize with the handling of the task through a short interactive session with feedback after every trial. The feedback consisted of the actual percentage *c*_*t*_ (using Eqs 1–3) they would have captured in trial *t* according their response *y*_*t*_ and our benchmark model. In addition, they were given a deviation score (mean squared error (MSE)) from the target percentage *δ*_*t*_ = (*c*_*t*_ − 0.65)^2^ ⋅ 1000.

In principle, a subject could learn how a pair consisting of observations ***d*** together with his response *y*, (***d***,*y*), relates to the capture probability *p* from experience in the 40 training trials. For a given learned mapping (***d***,*y*) → *p* he would have to adjust *y* such that *p* = 0.65. We regard this as unlikely for the following reasons. First, 40 trials do not provide a lot of data to learn from. Second, the mapping is high-dimensional and nonlinear which makes it hard to learn and susceptible to the specific instantiations of ***d*** across trials–as well as the choice of *y*. (***d***,*y*) and *p* are never simultaneously visible on the screen. And finally, batch learning requires memorizing all presented pairs which seems infeasible for participants. While on-line learning is possible, it typically suffers from slower convergence rates.

Participants could ask any questions to the experimenter prior to the experiment. The subsequent experimental session consisted of 320 trials with pauses together with feedback after every 5 trials. In the experiment, the feedback consisted of 5-trial averages of the quantities *c*_*t*_ and *δ*_*t*_ above that were computed since the last pause. Participants were supposed to minimize the deviation score and were payed more compensation when having a smaller deviation score to incentivize optimization. This supposedly promoted high motivation to prevent participants from resorting to computationally cheaper heuristic shortcuts. The task circumvents risk aversion since there is practically nothing that the participant can do to prevent losses other than stating the response as accurately as possible.

The bonus payment was determined by the mean of their final deviation score after removing the eight worst trials. The payment was determined by comparison to an array of five thresholds that were set according to the {0.1,0.2,0.3,0.4,0.5} cumulative quantiles of the empirical deviation score distribution across prior participants. A lower score corresponds to a better performance so that participants were payed an additional bonus of {5,4,3,2,1} € if their final deviation score was less or equal to the quantile thresholds. This is a relative way of rewarding their efforts to optimize their responses. Irrespective of their performance they were paid 10 € and hence on average received 11,50 € per session. The experiment was carried out with 23 participants. Later we excluded three of them because their behavior had little dependence on the stimulus.

The task was presented with Matlab Psychtoolbox 3.0.12. Participants made input with an USB-mouse that allowed them to precisely adjust the width of the response frame and confirm it with a click. Immediately after trial onset, they were presented with the dots and could start to expand/shrink the frame from a random initial width by moving the mouse up/down-wards. The points were visible throughout the entire time until the participant confirmed his response with a click. The program then either proceeded to the next trial or to the feedback/pause screen that indicates the averages over the five last trials of the percentage the participant would have captured as well as the numerical deviation score. In addition, information about the how many of all trials have already been completed was presented. The participant could proceed at his own pace.

### Computational models

We attempt to examine whether the behavior of the participants can be described by inference of probability distributions. More specifically, we attempt to infer whether their internal structural assumptions correspond to unimodal near-Gaussian distributions (Fig 2A) or might be better described by instance-based, nonparametric approaches (Fig 2B–2D) such as kernel density estimation. In addition, we checked whether selected heuristics can also account for the behavioral data.

#### Response mapping accounts for nuisance factors

Behavior is influenced by various factors and subjective assumptions of the participant which are difficult to model explicitly. Among these are subjective prior knowledge and probability distortion. Even for a probabilistic agent there exists some mathematical freedom as to what prior distribution over the latent variables to use. We did not explicitly include prior knowledge into our models but instead endowed the model with flexibility to approximately account for such effects.

We make use of the fact that ultimately, behavior such as the one derived from a probabilistic inference model just amounts to a specific mapping $d\to \hat{y}$ from inputs onto the response $\hat{y}$. Generally, for probabilistic models the mapping $d\to \hat{y}$ can be written in two steps. (i) Computing the sufficient statistic $\hat{S}$ which is then (ii) mapped onto the response, $d\to \hat{S}\to \hat{y}$, such as $\hat{S}=\sigma_{ML}(d)$ for the Gaussian. We use $\hat{S}$ to refer to any dispersion estimate and call $\hat{S}\to \hat{y}$ the response mapping. For non-probabilistic estimators, it just allows for additional tuning of the dispersion estimate. The introduction of the response mapping permits the construction of computationally simple models that may accommodate subjective knowledge of latent variables like *σ* in the second step.

This is illustrated in Fig 3A for the theoretical response curves (red, green). For maximum likelihood estimation (MLE) the response (red) is nothing but a linear mapping of the sufficient statistic *σ*_*ML*_(***d***) onto its output $\hat{y}$. The Bayesian benchmark model (green) also takes the sample size *N* = 4 and a uniform prior distribution over *σ* into account. Compared to MLE, its main effect is a bias of the responses towards intermediate values. The effect of a different prior on *σ* would merely manifest as a somewhat different mapping onto the response because *σ*_*ML*_(***d***) and *N* are sufficient statistics for *σ*. In other words, the model will produce the same results even when input ***d*** changes as long as the sufficient statistics remain the same. They compactly sum up all the information that is to be known about the hidden variables of a probabilistic model from the sample ***d***. Hence, distributions such as the posterior *p*(*σ*|***d***) or the prior *p*(*σ*) do not have to be explicitly represented in our model. Instead they are implicitly considered through the effects they exert on the response by allowing for additional freedom through a mapping. Apart from that, the mapping $\sigma_{ML}(d)\to \hat{y}$ also depends on the target percentage that the model is required to capture. A larger target percentage leads to a larger dependence on *σ*_*ML*_(***d***) and would e.g. manifest as a larger slope of the ML response (Fig 3A, red). The model may however account for the fact that participants suffer from probability distortion such that their internal target probability does not exactly match the one of a probabilistic agent (Eq 4).

The response mapping from the dispersion estimate to the response, $\hat{S}(d)\to \hat{y}$, is chosen to be the same for all models and is intended to be flexible enough to account for these implicit effects. Empirically we found that a quadratic polynomial is only minimally better than a linear mapping (using the weighting-model, below). The improvements on the group level are significant (increased median cross-validation log likelihood (CVLL) across participants, Wilcoxon sign rank test, *p* = 0.0027) but small in absolute terms (median CVLL difference 3.66 dHart, 95% CI (0.34, 7.15) dHart, below). For this weak nonlinearity and to obtain a sparse model formulation, we consider a polynomial of first order to be a sufficiently good approximation to represent the response mapping.

$$\hat{y}=\beta_{0}+\beta_{1}\hat{S}(d)$$ (5)

The models that we consider differ only in how they compute the dispersion measure $\hat{S}$. They may introduce additional parameters which are detailed below. We start by describing approximative models that do not make use of distributions first. We will explicitly consider heuristic models. In general, heuristics are not linked to optimal responses in a principled way but nevertheless might yield satisfactory results. Every estimator that correlates with *σ*_*ML*_ contains some useful information about the dispersion and may thus be used. As heuristics are frequently associated with less effortful processing, we consider simple and visually salient quantities that may be readily assessed by the participants. As another approximate model, we test a weighting model that emphasizes certain stimulus features. We will then describe probabilistic models that derive responses from different distributional estimates and conclude with a predictive model intended to serve as an estimator for the upper bound on predictability given our data.

#### Maximum model

This model uses the distance of the point that is farthest away from the center, that is, $\hat{S}=max(\left|d\right|)$. This function can be considered a simple heuristic approach because it reduces the input information to be processed, but as this distance strongly correlates with *σ*_*ML*_ it is expected to be predictive of behavior.

#### Range model

This model uses an estimate of dispersion based on the difference between the leftmost and rightmost point $\hat{S}=\max(d)-\min(d)$. Again, this quantity is correlated with *σ*_*ML*_.

#### Weighting model

The maximum likelihood estimator ***σ*_*ML*_** can be generalized in that it assigns different weights to individual points when calculating the root mean square deviation. The observations ***d*** are indexed according to their excentricity, i.e. their absolute deviation from zero such that |*d*_*n*_| ≥ |*d*_*m*_| for *n* > *m*.

$$\hat{y}(d)=\beta_{0}+\hat{S}(d)=\beta_{0}+\sqrt{\frac{1}{N}\sum_{n=1}^{N}\omega_{n}d_{n}^{2}},\;\omega_{n}\geq 0$$ (6)

The parameter *β*_1_ of the response mapping $\hat{y}=\beta_{0}+\beta_{1}\hat{S}$ (Eq 5) is factored into the *ω*_*n*_ and set to one to avoid under-constrained solutions for regression. We may enforce the summation constraint, ∑_*n*_*ω*_*n*_ = *N*, on the weights after fitting to interpret the weights as relative contributions with respect to the case of *ω*_*n*_ = 1, which corresponds to inference of a Gaussian. This can be done by factoring out a term $\sqrt{N/\sum_{n}\omega_{n}}$ which can be formally assigned to *β*_1_. We consider the equal weighting of the square of each point’s position $\sigma_{ML}=\sqrt{1/N\sum_{n=1}^{N}d_{n}^{2}}$ a non-trivial pattern of inference of a normal distribution.

Within this model, we also test the heuristic of considering just one out of all *n* = 1,…,*N* points, $\hat{S}(d)=|d_{n}|$. In this case, just one of the four weights should be four while the others will become zero due to the summation constraint. The task is constructed such that the position of the third most excentric point closely corresponds to the target percentage. Yet, we found that this heuristic is evidently exploited by just one participant (normalized *ω*_3_′ = 0.95, *d*_3_ almost explains full variance, *R*^2^ = 0.96).

Because of the generality and the computational ease with which optimization can be performed for this model, we use it to test variants of the response mapping Eq 5. We test whether participants behave in accordance to a prior belief about the range of dispersions across trials. A pure ML approach ignores prior knowledge and leads to responses proportional to the dispersion estimate $\hat{S}(d)$ (Fig 3A, red). If that was sufficient to predict behavior, a model whose output is restricted to be proportional to the dispersion estimate (omitting constant term in Eq 5) should perform equally well.

$$\hat{y}(d)=\hat{S}(d)=\sqrt{\frac{1}{N}\sum_{n=1}^{N}\omega_{n}d_{n}^{2}}$$ (7)

Likewise, a model which additionally features a quadratic term $\hat{y}=\beta_{0}+\hat{S}+\beta_{2}\hat{S}$ is used to test for the nonlinearity of the response mapping. The weighting model is chosen for these tests as it can flexibly account for other systematic biases in behavior that are not related to prior knowledge.

#### Normal model

Making inference using a normal distribution is equivalent to the mapping $d\to \hat{S}\to \hat{y}$ in which $\hat{S}=\sigma_{ML}$ is the sufficient statistic and the MLE of the Gaussian. To match the responses of our benchmark model, the response mapping $\hat{S}\to \hat{y}$ must equal the green curve in Fig 3A. The chosen response mapping for regression (Eq 5) can only provide a linear approximation to this curve but was chosen based on considerations regarding model sparsity and the empirical evidence to be sufficient to capture behavior.

#### Generalized normal model

The dart metaphor and the task instructions suggest that the distribution of darts follows some symmetric and bell-shaped curve centered at zero. As a perfect match between the true and assumed distributions by the participants is not expected, we consider a generalized normal distribution which has an additional shape parameter *p* > 0 so that it can represent a family of distributions.

$$p(x|\mu ,\alpha ,p)=\frac{p}{2\alpha \Gamma (1/p)}\exp\left[-{\left(|x-\mu |/\alpha \right)}^{p}\right]$$ (8)

It effectively generalizes the exponent of the normal distribution for which it takes a value of *p* = 2. For small *p*, the distribution is more peaked whereas it approximates a plateau like distribution for larger values (Fig 1B). We assume that the exponent parameter *p* is constant across trials and treat it as an additional fitting parameter. For a known mean of zero, μ = 0, the maximum likelihood estimator for *α* is $\sqrt[{p}]{p/N\sum_{n=1}^{N}{\left|d_{n}\right|}^{p}}$ which we identify with the dispersion estimate $\hat{S}$. In the limit of *p* → ∞ it corresponds to the heuristic maximum model above. We also tested a generalized normal model which infers *μ* on a trial-by-trial basis for a given exponent *p* to test whether dropping the assumption of a centered distribution can better explain behavior. In this case, Eq 4 is explicitly solved, and its result is assigned to $\hat{S}$. As it was found to be worse than the centered normalized distribution on the group-level (exceedance probability *p*_*exc*_ > 0.999), we chose to only report results using a centered distribution.

#### Gaussian kernel density estimation model

If one imposes only minimal structural constraints, more freedom is given to the data to determine the inferred density. One may assume that even small samples represent the population well and that future observations will cluster around the already observed instances. One way to do so is to estimate *p*(*x*|***d***) over future events *x* based on a kernel method. It generalizes observed data points *d*_*n*_ by assigning probability density proportional to a kernel function *k*(*x*,*d*_*n*_) to their vicinity and thus constitutes a data smoothing problem (Fig 2D). For the whole training set ***d***, kernel density estimation centers a kernel on each observation and sums up their contributions to determine *p*(*x*|***d***) as:

$$p(x|d)=NP(x|\eta ,d_{1},\ldots ,d_{N})=\frac{1}{N}\sum_{n=1}^{N}k(x|d_{n},\eta )$$ (9)

It is a nonparametric method because it does not assume a certain parameterized family of probability distributions for *p*(*x*) apart from the kernel. The kernel function *k* typically decays with the distance between *x* and *d*_*n*_. Here we assume that it has the shape of a normal distribution *k*(*x*|*d*_*n*_,*η*) = *N*(*x*|*d*_*n*_,*η*). The kernel width *η* = *η*(***d***) is in principle a free parameter but needs to be sensibly chosen with respect to the dispersion of the data. Manual testing revealed that *η* = *a* ⋅ (*d*_3_ + *d*_4_)/2 is a reasonably good approximation to the unknown *η*(***d***) function. Thus, potentially even better performance might be achievable than the one reported here. The model’s dispersion estimate, $\hat{S}$, regarding the 65% capture probability is determined by inserting the inferred distribution Eq 9 into Eq 3 and then solving Eq 4.

In the limit of vanishing kernel widths *η* → 0 (*δ*-distributions) the response for the target percentage of *p*_*t*_ = 0.65% converges to the third most excentric point. We refer to this approach as *δ*-KDE (Fig 2C). In this limiting case, one would merely capture the target fraction *p*_*t*_ of observed points on the screen, thus replacing an estimation of the target fraction *p*_*t*_ of the population with a corresponding estimation of *p*_*t*_ on the sample.

**Tiling model.** To capture a certain percentage of points of the sample, one must have some sort of quantile function that outputs the region containing the desired percentage. Explicit density models such as KDE entail a quantile function. A simple alternative is to construct some normalized histogram. We attempt to do so with the constraint that an observation point only exhibits a local effect on the constructed density (Fig 2B). Specifically, the contribution to the overall density of one data point only depends on its own position and on the position of its adjacent points.

More formally, this can be achieved by tiling the space between observations into rectangular, adjacent but non-overlapping basis distributions. We adhere to the additional constraint that the *N* ordered points correspond to the (0.5/*N*,1.5/*N*,…,(*N* − 0.5)/*N*) cumulative quantiles. Hence each basis distribution spanned between points has to be normalized by *N*. To assign the remaining probability 0.5/*N* below the lowest point *d*_1_ we use a uniform distribution U(*d*_1_ − *d*_2_,*d*_1_) whose support equals the distance to its only adjacent point *d*_2_ (and likewise for the largest point). Representations of probability densities based on orthogonal basis functions are suggested as a solution to tractably represent complex densities [6].

**Gaussian process regression.** Gaussian Process Regression (GPR) [49] is used to estimate the upper bound on predictability of the participants’ behavior. It does not lend itself readily to an interpretation of how participants solve the problem on a given trial. It is however very flexible and successful in prediction by exploiting consistency between input ***d*** and output *y* across pairs of trials (*i*,*j*). We used GPR since it is a bias free estimator of the distribution *p*(*y*|***d***) which is assumed to be normally distributed with a constant intrinsic noise parameter *σ*_*I*_. We chose a Gaussian kernel function

$$k(d_{i},d_{j})=\theta \cdot \exp[-\frac{1}{2}\sum_{n}{\left(d_{in}-d_{jn}\right)}^{2}/\sigma_{n}^{2}]$$ (10)

that defines a scalar measure of similarity and the entries of the covariance matrix of the GP as $C_{ij}=C(d_{i},d_{j})=k(d_{i},d_{j})+\sigma_{I}^{2}\delta_{ij}$. Input pairs (***d***_*i*_,***d***_*j*_) that are considered similar in this sense should result in comparable responses (*y*_*i*_,*y*_*j*_) if the process *p*(*y*|***d***) is consistent. Prediction is more strongly influenced by those trials’ responses *y* for which (***d***_*i*_,***d***_*j*_) are similar. To make predictions for a new input ***d***_*ν*_, we evaluate the mean of the predictive distribution $\hat{y}(d_{\nu })=k^{T}C^{-1}y$. Here ***k*** has the entries *k*(***d***_*i*_,***d***_*ν*_) with *i* indexing all trials in the training data. Likewise, *C* and ***y*** are constructed from all the training data used to derive predictions. For each trial ***d***_*t*_ = (*d*_*t*1_,…,*d*_*tN*_), symmetry is exploited by sorting the points in ascending order of excentricity. To set the hyperparameters of the GP, (*θ*,*σ*_1_,…,*σ*_*N*_,*σ*_*I*_), its generalization error is minimized. To do so, the mean of the test sets of Eq 13 of a 5-fold cross validation (CV) procedure is calculated. This procedure is part of training the GPR. We also attempted to predict behavior using a simple 1-hidden-layer feedforward neural network. Despite being a successful predictor, its performance was inferior to the GPR which is why we chose to only report the latter.

**Baseline model.** The baseline model is chosen to provide a simple lower bound estimate for predictability that is independent of the trial-by-trial variations of the stimulus. This model calculates the mean of the responses of all its input ***y***_*in*_ (training set). It thus makes the same prediction on every trial *t*.

$${\hat{S}}_{t}=\langle y_{in}\rangle$$ (11)

**Inter-trial and feedback dependence.** We investigated the influence of other quantities on behavior that participants might have (erroneously) utilized to guide their responses. To test for a dependence on the preceding trial, the estimator $\hat{S}$ is chosen to be the previously stated response.

$${\hat{S}}_{t}=y_{t-1}$$ (12)

There is a significant effect with respect to baseline (exceedance probability, *p*_*exc*_ > 0.99), yet the effect on behavior is virtually negligible as the overall predictive performance is very low (median cross-validation log likelihood across participants −318 dHart, 95%-CI (−356,−300) dHart, with respect to the best model for each participant). The influence of the previously presented feedback about the capture percentage is similarly tested but its effect is found to be even weaker (−327 dHart, 95%-CI (−368,−312) dHart). Together with the evidence that participants did not adjust closer to the target capture percentage of the task (Fig 4B), we consider it unlikely that feedback affected behavior to a considerable extent.

**Overview of model parameters.** The models used have a different number of parameters depending on the dispersion estimate $\hat{S}$. The ones reported in the main text are summarized in Table 1.

**Table 1:** Overview of model parameters.

Model	Abbreviation	Fitting parameters					
**Maximum**	max	*β*_0_	*β*_1_				
**Range**	rng	*β*_0_	*β*_1_				
**Weighting**	wgt	*β*_0_	-	*w*_1_	*w*_2_	*w*_3_	*w*_4_
**Normal**	nm	*β*_0_	*β*_1_				
**Generalized normal**	gnm	*β*_0_	*β*_1_	*p*			
**Kernel density estimation**	kde	*β*_0_	*β*_1_	*a*			
**Tiling**	tlg	*β*_0_	*β*_1_				
**GPR**	gpr	Nonparametric, hyperparameters: (*θ*,*σ*_1_,…,*σ*_*N*_,*σ*_*I*_)					

**The response distribution.** The probability of obtaining the response *y*_*t*_ on trial *t* conditional on the data ***d***_*t*_ and the model parameters is assumed to be a mixture distribution of two contributions. The first and dominant term is a normal distribution centered on the model prediction ${\hat{y}}_{t}$, modeling task-intrinsic noise around the estimates. Upon preliminary inspection of the data we found considerable heteroscedasticity with higher response variability for larger sample dispersions.

To take this feature of the response data into account, we assume that the standard deviation (SD), *θ*, of the distribution over response *y*_*t*_, $N(y_{t}|{\hat{y}}_{t},\theta ({\hat{y}}_{t}))$, is a function of the model output ${\hat{y}}_{t}$. The model output is denoted by $\hat{y}$ to distinguish it from the response *y* of the participant which is formally represented by a draw from the response distribution to account for behavioral variability. Instead of assuming a parametric relationship and the need of further parameters to be fitted in the model, we make a parameter free estimate by assuming a discretized function, as follows. We divide the whole model output $\hat{y}$ into *Q* equally filled quantiles *q* ∈ {1,…,*Q*} by assigning trial *t* to quantile *q*_*t*_. For every quantile *q*, the SD is estimated separately by calculating $\theta_{q}={\left(\sum_{j}{\left(y_{j}-{\hat{y}}_{t}\right)}^{2}/J\right)}^{1/2}$ (*j* = 1,…,*J* indexes trials belonging to quantile *q*). Hence, whenever there is heteroscedasticity, the true function $\theta (\hat{y})$ is approximated by the estimated bin values. For homoscedasticity all *θ*_*q*_ are the same and collapsing bins would make no difference. The resolution of the function is higher when many quantile divisions are used provided the *θ*_*q*_ can still be estimated faithfully. We consider *Q* = 5 a suitable choice for our problem.

As our data might be contaminated by processes other than dispersion estimation, such as lapses, we take precaution against far outlying responses. We calculate a trimmed standard deviation, i.e. before calculating *θ*_*q*_ we remove values below or above two interquartile ranges from the lower or upper quartile respectively. However, this applies to *θ*_*q*_ estimation only. No points are removed from calculating the response likelihood

$$p(y|d_{1},\ldots ,d_{T})=\prod_{t=1}^{T}(1-\epsilon )N(y_{t}|{\hat{y}}_{t},\theta_{q_{t}})+\epsilon .$$ (13)

Additionally, to prevent isolated points from being assigned virtually zero probability, we generally add a small probability of *ϵ* = 1.34 × 10^−4^ to all. This corresponds to the probability of a point at four standard deviations from the standard normal distribution. For non-outlying points this alteration is considered negligible.

**Estimating model evidence.** The evidence that each participant’s data lends to each model is derived from predictive performance in terms of the cross-validation log likelihood (CVLL). For training, we maximized the logarithm of the response likelihood (Eq 13). To maximize the chances of finding the global maximum even for non-convex problems or shallow gradients, every training run first uses a genetic algorithm and then refines its estimate with gradient based search (MATLAB ga, fmincon). The CVLL for each participant and model is summarized by the mean of the logarithm of the response likelihood (Eq 13) on the test set across all cross validation (CV) folds.

As cross validation is a computationally expensive method, we use a random 5-fold split of data into training and test sets such that each training point is used four times for training and once for testing. However, to make splits more representative of the sample we use a stratified version of CV by ensuring that the mean target variable is approximately equal in all folds. This is done by assigning data points to one of the 8-quantiles of the distribution of the target variable. We constructed slices that contain one value from each quantile. Subsequently, we sampled strata to create the 5-fold CV splits. To improve the reliability of per participant estimates of the model evidence (CVLL) we repeated this procedure with different random splits and aggregated the output so that in total 10 CV splits are performed for each participant and model.

Differences in model evidence, Δ, are reported on a log-scale in decibans (also decihartleys, abbreviated dHart) that may be used to interpret the significance of the results of individual participants. According to standard conventions, we consider a value of 5 > Δ barely worth mentioning, 10 > Δ ≥ 5 substantial, 15 > Δ ≥ 10 strong, 20 > Δ ≥ 15 very strong and Δ ≥ 20 decisive.

**Group level comparison.** Instead of making the assumption that all participants can be described by the same model, we use a hierarchical Bayesian model selection method (BMS) [50] that assigns probabilities to the models themselves. This way, we assume that participants may be described by different models. That is a more suitable approach for group heterogeneity and outliers which are certainly present in the data. The algorithm operates on the CVLL for each participant (*p* = {1,…,*P*}) and each model (*m* = {1,…,*M*}) under consideration and estimates a Dirichlet distribution Dir(***r***|*α*_1_,…,*α*_*M*_) that acts as a prior for the multinomial model switches *u*_*pm*_. The latter are represented individually for each subject by a draw from a multinomial distribution *u*_*pm*_ ~ Mult(1,***r***) whose parameters are *r*_*m*_ = *α*_*m*_/(*α*_1_+…+*α*_*M*_). We use the CVLL and assume an uninformative Dirichlet prior ***α*_0_** = **1** on the model probabilities. Later, for model comparison, exceedance probabilities, $p_{exc}=\int_{0.5}^{1}\mathrm{Beta}(\alpha_{i},\sum_{j\neq i}\alpha_{j})$, are calculated corresponding to the belief that a given model is more likely to have generated the data than any other model under consideration. High exceedance probabilities indicate large differences on the group level. We consider values of *p*_*exc*_ ≥ 0.95 significant (marked with *) and values of *p*_*exc*_ ≥ 0.99 very significant (marked with **).

## Supporting information

S1 Dataset Participants’ experimental data. All data used for the analysis is available as a Matlab data file.
(MAT)
